# What's Out There Making Us Sick?

**DOI:** 10.1155/2012/605137

**Published:** 2011-10-24

**Authors:** Stephen J. Genuis

**Affiliations:** Faculty of Medicine, University of Alberta, 2935-66 Street, Edmonton, AB, Canada T6K 4C1

## Abstract

Throughout the continuum of medical and scientific history, repeated evidence has confirmed that the main etiological determinants of disease are nutritional deficiency, toxicant exposures, genetic predisposition, infectious agents, and psychological dysfunction. Contemporary conventional medicine generally operates within a genetic predestination paradigm, attributing most chronic and degenerative illness to genomic factors, while incorporating pathogens and psychological disorder in specific situations. Toxicity and deficiency states often receive insufficient attention as common source causes of chronic disease in the developed world. Recent scientific evidence in health disciplines including molecular medicine, epigenetics, and environmental health sciences, however, reveal ineluctable evidence that deficiency and toxicity states feature prominently as common etiological determinants of contemporary ill-health. Incorporating evidence from historical and emerging science, it is evident that a reevaluation of conventional wisdom on the current construct of disease origins should be considered and that new knowledge should receive expeditious translation into clinical strategies for disease management and health promotion.

An analysis of almost any scientific problem leads automatically to a study of its history.

—Ernst Mayr

## 1. Introduction

Greek philosophers including Thales and Aristotle sought logical, sensible, and cogent explanations for the spectrum of human experience, for everyday events, and for the way the world works [1]. From the times of ancient Greek civilization, through the middle ages, and into our current technological age, thinkers and scientists have pondered and sought answers as to why people get sick. From its fundamental genesis in philosophy, with emphasis on skepticism and critical thought, modern medical science has emerged with conclusions about the etiology of suffering and disease. 

In this paper, a few snapshots of medical history illuminating the origins of illness will initially be presented. Through the lens of history and emerging science, current conventional wisdom about disease etiology will then be examined. Finally, evidence in disciplines such as molecular medicine, epigenetics, and environmental health will be explored to explain the root cause of chronic and degenerative disease, a problem that afflicts so many in the world today.

## 2. Historical Perspective on Disease Etiology

### 2.1. The Origins of Modern Medicine

As with every culture including the present, the ancient Greeks believed that they embodied the ultimate in sophistication [1]. As world leaders of progressive thought in philosophy, education, and science, academics in early Greece assumed that events of life including calamity and illness were the result of metaphysical forces and mystical powers. Apollo would send, it was thought, his invisible arrows to inflict pain and suffering on the condemned. 

Amidst this milieu, however, a young Greek physician named Hippocrates challenged the popular paradigm that supernatural factors were the driving force behind disease [2]. With a skeptical mindset, Hippocrates scrutinized conventional medicine of the day, he challenged disease attribution to paranormal factors, and he rejected the accepted medical standard of care—appeasement of mystical forces with chemical concoctions. Searching for rational evidence to explain origins of illness that could be demonstrated through reproducible observation and experimentation, Hippocrates endeavored to convert the field of medicine from a religion to a science [2]. 

Witnessing health demise after some patients consumed dispensed poisons from bribed practitioners, he penned the Hippocratic Oath to challenge the ethics of corrupt physicians [3]. With observation of divergent population health in differing locales, dissimilar individual constitutions from birth, and variations in health related to diet and sun exposure, Hippocrates concluded that nutrition, inborn factors, and environmental influences were major determinants of sickness and health [2]. Building on the fundamental scientific premise that every effect has to have a source cause, he surmised that perhaps if the cause of illness was found, then disease might be cured. Writing in the Hippocratic Corpus, this young physician and his followers defied both the spirit and the practice of metaphysical traditional medicine.

#### 2.1.1. The Early Years

The basics of science—vigilant observation, empirical experimentation, and reproducible research—were brought into the ethos of medical practice, a monumental accomplishment which earned Hippocrates the worthy title of “Father of Modern Medicine” [4]. Although some of his interpretations were primitive and misguided, the substantive basis of his scientific approach to understanding the etiology of illness remains credible to this day. He came to believe that disease commences because of a cause, disease persists because the cause persists, and that disease can only desist when the cause desists [2].

Notable scientists in early Common Era centuries continued to observe and explore causes of disease. Galen (circa 130–200 AD), for example, spent his early career doctoring gladiators and noted that those with wounds often became ill and frequently succumbed. Hypothesizing that wounds provided “windows to the body,” Galen deduced that unhealthy vapors rising from the ground formed poisonous gases which entered through wounds to cause illness [5]. Although various theories and ideas emerged over the next few centuries, limited original contribution relating to disease causation was recorded until the Middle Ages [4, 5]. 

Throughout the early centuries, however, the metaphysical construct of disease causation, a mindset engrained in the fabric of many cultures, continued to pervade medical practice. Some afflicted individuals, for example, were executed as demonic possession was often considered the source of mental illness and aberrant behavior. Black Death, the plague which consumed countless lives in the fourteenth century, was oft blamed on the Jews—an attribution which spurred violence and prompted reigning Pope Clement VI to issue an edict pronouncing a misalignment of Mars, Jupiter, and Saturn as the true culprit. Commencing in the 16th century, however, a number of notable discoveries confirming Hippocrates' notion about natural causes of illness began to emerge.

**(1) Toxicant Exposures**

Immortalized as Paracelsus, the “Father of Toxicology,” Auroleus Phillipus Theostratus Bombastus von Hohenheim worked in the 1500s as an alchemist, astrologer, and physician. With the observation that use of chemicals such as mercury and opium could change the mental and physical status of individuals, Paracelsus introduced the idea that disease was the result of a chemical imbalance [6]. With much experimentation, he pioneered the use of elements and chemical compounds in medicine.

Treated as an outcast and heretic by the established medical community, Paracelsus noted that, at low dose, certain compounds appeared to be therapeutic, while at larger dose they acted as poisons [6]. The emergence of medicine by alchemy increasingly became the standard of care with assorted toxic elements including mercury, lead, and arsenic being used by practitioners to deal with myriad afflictions from fatigue to syphilis.

Paracelsus affirmed Hippocrates' observation, however, that chemical toxins had the potential to act as a poison and to induce illness if a threshold dose was exceeded. Paracelsus' defining publication, *On the Miners' Sickness and Other Diseases of Miners,* documented occupational risks associated with exposures during metalworking [7]. In conclusion, exposure to chemical toxins was identified as a cause of sickness and death.

**(2) Nutritional Deficiency**

A major breakthrough in medicine occurred on the high seas with the British Royal Navy. Initially described by Hippocrates more than two thousand years ago, a disease called scurvy consumed many passengers and crew on long-distance voyages. A Scottish surgeon, Dr. James Lind, puzzled as to why some of his crew would succumb to this treacherous disease while others did not. Wondering whether dietary habits might be a factor in illness, Lind prescribed different diets for individuals deteriorating with scurvy, and, as described in his 1753 book, *A Treatise of the Scurvy,* he found that citrus fruit rapidly and consistently cured this previously fatal malady [8].

But as consistently occurs in medicine when new ideas and scientific discoveries regarding disease causation are offered—no matter how compelling the evidence—Lind's findings were initially mocked and disregarded. Only after decades passed did the British navy and the medical world at large accept his evidence in order to stop the flood of needless scurvy deaths. In conclusion, deficiency of some essential nutrient or nutrients was recognized as a cause of sickness and death.

**(3) Genetics**

Initially described as a monster for his findings, Austrian monk and scientist Gregor Johann Mendel observed evidence of logical transmission of inherited traits from one generation to the next in his experiments with pea plants. Mendel, subsequently titled the “Father of Modern Genetics,” repeatedly demonstrated in the nineteenth century that inheritance patterns were consistent and followed particular laws [9].

Although Mendel's work was initially met with disdain and rejection, subsequent research after his death demonstrated a logical bond that transmitted through generations, not only in plants but also in the animal kingdom. Mendel's findings spurred further study and eventually became the foundation of modern genetics—a discipline which has repeatedly confirmed “genetic predisposition” as an important factor in the causation of illness.

**(4) The Germ Theory**

One of the most remarkable discoveries contributing to the discourse on disease etiology relates to the finding of pathogens or disease-causing germs [4]. At a time when more than 20% of women died in childbirth, a young Hungarian obstetrician named Ignaz Philipp Semmelweis noted that impoverished women delivering outside of hospitals had a maternal mortality rate only a fraction of that for women receiving hospital care. Also observing that maternal death rates plummeted when medical students were absent and birthing was assisted by midwives, Semmelweis comparatively investigated approaches by students and midwives [10].

Noting that medical trainees proceeded from anatomy labs to obstetric suites, he hypothesized that some pathogenic agent may be carried to the maternity area and thus introduced a hand washing technique. When maternal deaths precipitously fell overnight, this pioneer realized that he had uncovered the cause of puerperal fever. Careful documentation of evidence and desperate appeals to colleagues to replicate his work only evoked scorn and contempt.

Witnessing sickness and death from infection complicating surgery or open fractures, work in the nineteenth century by French chemist and microbiologist Louis Pasteur, “Father of the Germ Theory,” added to mounting evidence of the link between microbial agents and sickness [11]. Along with other pioneers in microbiology, including Ferdinand Cohn and Robert Koch, it became apparent in the late 19th century, that pathogens were a common cause of sickness—a realization that provoked a temporary shift in conventional wisdom whereby the causation of most disease was attributed to germs [4].

**(5) Psychological Determinants**

During the 19th and early 20th centuries, Sigmund Freud, Carl Young, Abraham Maslow, Ivan Pavlov, and other innovators theorized at length on psychological mechanisms leading to ill-health [12]. Although many of the specific mechanisms proposed such as Freud's theory of psychosexual stages of development are now in question, the idea that psychological pathology can contribute to ill-health has repeatedly been confirmed. More recent laboratory study has found dramatic changes in physiological parameters and indices in response to psychological states, leading the medical community to accept disordered psychology as a potential source of sickness.

#### 2.1.2. Historical Overview of Disease Causation

**Figure 1:**
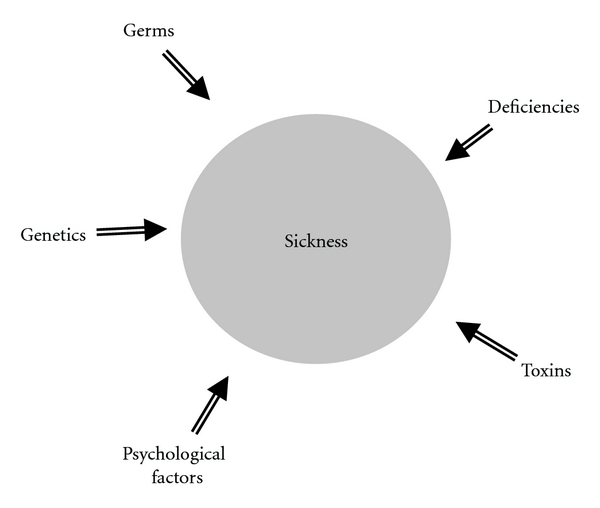
Sum total of etiological determinants of illness.

There are many other notable heroes in medical history who have contributed to the understanding of health and disease [4, 5]. For example, Christiaan Eijkman won the Physiology and Medicine Nobel Prize in 1929 for his discovery, at a time when everyone was looking for a germ, that beriberi resulted from deficiency of an essential nutrient (thiamine) absent in the polished white rice of European settlers stationed in the Orient [13]. On careful analysis, however, each of the other findings and discoveries on disease etiology represented further developments and clarifications on these five determinant themes—nutritional deficiency, toxic exposures, genetic predisposition, infectious agents, and psychological dysfunction (Figure 1). These five pillars of disease etiology have repeatedly been demonstrated historically to be the source of all illness. So how does modern medicine view the etiology of illness in view of this body of accumulated historical science?

## 3. Contemporary Beliefs about Disease Etiology

**Figure 2:**
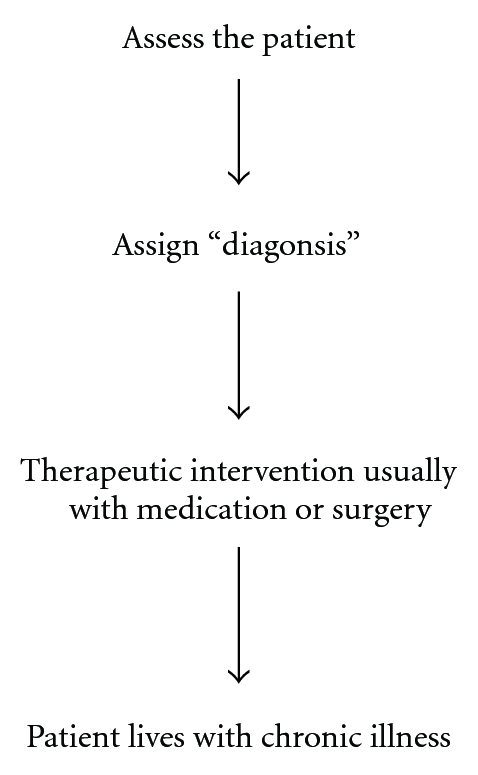
Common algorithm for management of contemporary chronic illness.

To best determine how contemporary medicine views the origins of illness, it is instructive to observe how mainstream medicine is practiced and to explore underlying assumptions. A typical algorithm (Figure 2) is used when patients with chronic disease visit their physician [14], an approach which reflects clinical practice guidelines—pervasive administrative directives used to guide the actions of individual physicians [15]. 

Through an interview, physical examination, and laboratory testing, the physician does an assessment in order to determine the appropriate “diagnosis”—a label which indicates that the patient's signs, symptoms, and laboratory results match or fulfill common criteria for that label [16]. After diagnosis has been assigned, it is common for intervention to commence, frequently employing medications or surgery. For chronic conditions, which now form the overwhelming burden of illness globally, patients usually persist with therapy indefinitely to cope with their sickness. But what does this algorithm tell us about prevailing assumptions regarding the cause of sickness?

### 3.1. Predestination Construct

As the diagnosis does not assign any source cause or reason for the development of the condition, a search for cause in this algorithm remains neglected. As deliberate neglect of disease causation might be considered remiss and unscientific, why is etiology not actively pursued as a fundamental step in the approach to sick patients? 

**Figure 3:**
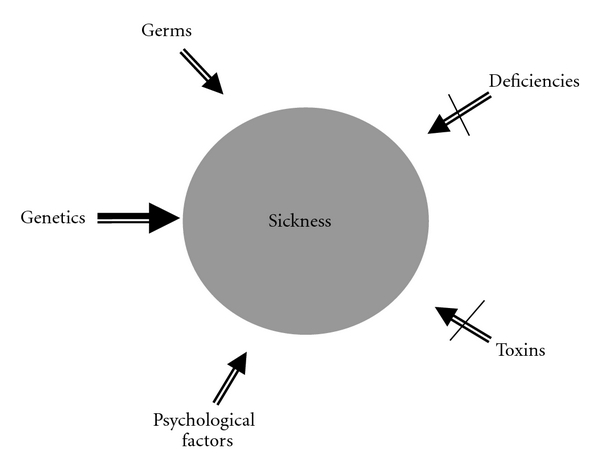
General perception in contemporary clinical practice about common etiological determinants of chronic illness in the Western World.

Most practitioners assume that, other than situations of infection or psychological compromise, source etiology of most chronic illness reflects genetic fate—the idea that people are predestined as hapless victims in a cosmic game of genetic roulette. This genetic predestination paradigm leaves no alternative but to provide drugs and surgery to overcome the misfortune of having the wrong parents [17]. Through a historical lens, however, this contemporary fatalistic approach disregards the deficiency and toxicity components as common causative factors in disease (Figure 3) [18]. 

Five fundamental observations in recent science and epidemiology literature, however, have begun to challenge the inherited or genetic predestination paradigm.

- Identical twins with the same genome frequently have different health outcomes [19].
- Many people develop chronic conditions that are absent in their ancestry.

(iii) While genomes have not changed, rates of various chronic afflictions including autism, depression, dementia, and some types of cancer have escalated considerably.

(iv) Geographic differences for various chronic diseases are evident [20].

(v) Disease incidence among population groups often changes significantly with migration and adoption of new lifestyles [21].

It is unlikely that genetics accounts for the more than 2500% increase in autism [22] or the profound increase in hysterectomies performed over the last 25 years [23]. Genetics is not likely to account for the notable increase in heart disease and diabetes among Japanese immigrants settling in America or the increased likelihood of acquiring autoimmune disease for populations residing in northern latitudes. Some have attributed the prevalence of chronic illness simply to an aging population, but the recent escalation of chronic disease in pediatric populations [24] refutes this misconception. While genetics may predispose to illness, deductive reasoning suggests that other factors must be influencing health status.

## 4. Why Are People Getting Sick?

### 4.1. Molecular Medicine: Genomics

Recognizing that the human organism is fundamentally a community of specialized cells made up of countless molecules, the scientific discipline of molecular medicine endeavors to gain insights into the genetic, molecular, and cellular bases of disease. The human genome project has confirmed that each of us is unique genetically, and thus our biological functioning at a molecular level is not identical [25]. This breakthrough has spawned the expanding field of genomics. 

The way we respond to our environment, medications, and stressors will depend on our specific genetic imprint. Accordingly, broad-based conclusions on the efficacy of certain treatments may be less than reliable when applied to specific individuals—each individual with a distinct biochemistry will respond differently to each medication based on their genetic makeup. The fields of pharmaco-genomics and nutrigenomics have recently emerged where medication and nutrient interventions are personalized and tailored to the specific genomic state of the individual [26, 27]. 

With new laboratory investigations, the unique genetic and biochemical makeup of the individual can be assessed in order to determine irregularities at a molecular level that may be influencing health. Individual genomic assessments may provide evidence for predisposition to various afflictions. BRCA1, for example, is a genetic marker which may indicate predisposition to breast cancer [28]. The expanding repertoire of genetic markers confirms that genetic predisposition to sickness is a scientific reality. With the inability to modify human genes thus far, however, our genetic map is fixed and thus our predisposition to sickness is immutable. There is another force that will be discussed, however, which appears to control whether our predisposition to a specific sickness will manifest as disease or remain quiescent and manifest as health.

### 4.2. Molecular Medicine: Environmental Health Sciences

A recent edition of *Science* highlighted the emerging reality that “chronic illness is the consequence of inherited diversity of the genetic code combined with environmental biochemical influence” [29], while the Centers for Disease Control and Prevention recently concluded that “virtually all human diseases result from the interaction of genetic susceptibility and modifiable environmental factors” [30]. Ongoing scientific research has repeatedly confirmed that various modifiable factors within the environment of our body have the ability to interact with our genetic predisposition to cause sickness. 

One way that environmental factors cause illness is through gene regulation. A discipline within the field of molecular medicine called epigenetics endeavors to study factors and identify determinants which regulate and control the expression of genes [31]. In other words, science is demonstrating that genes are not autonomous structures which determine fate but rather are molecules which respond to and are often regulated by modifiable environmental triggers. A loaded gun will not cause damage unless triggered; a vulnerable gene may remain dormant unless triggered by specific factors within the terrain of the body. The impact of epigenetic environmental influences makes genetic expression a dynamic reality with new evidence demonstrating the potential to transmit adverse genomic expression and clinical pathology through generations [32, 33].

The study of environmental health sciences or environmental medicine is the clinical application of molecular and epigenetic medicine. It allows for the study of the modifiable environment in order to identify and correct abnormalities that are triggering sickness. But what are these modifiable factors in our environment that have the ability to interact with genes to cause sickness?

### 4.3. The Blind Spots

**Figure 4:**
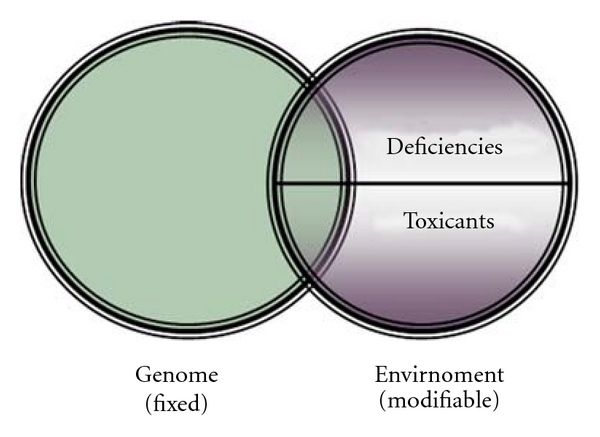
Etiology of illness.

Broadly speaking, there are only two factors in the environmental sphere: (i) requirements—are we getting what we need in order to thrive; and (ii) toxins—are we free of adverse influences. Evidence continues to accumulate that the main environmental determinants of illness are deficiency and/or toxicity states interacting with a fixed genome (Figure 4) [34]. In other words, if we are missing essential components that our human body requires in order to function, illness results; if there are adverse factors obstructing or interfering with normal biological function, illness results. This principle is eminently sensible as it applies to all machines as well as plant life. If a plant is to thrive, certain essential requirements are required, all of which are requisite to plant survival. If specific toxicants are introduced, the plant may wither.

Observing through the lens of history, these two determinants of health and disease are precisely the two areas neglected in much of contemporary medical practice (Figure 3). Why have deficiency and toxicity concerns, domains so clearly and repeatedly identified in medical history as causative in illness, been virtually disregarded in much of present-day conventional medicine?

### 4.4. Deficiency States as a Cause of Illness

Nutrient biochemicals are the building blocks of our human frame and the necessary prerequisites for ongoing physiological function [35]—we are a collection of biochemicals. Using nutrient raw materials, our body manufactures all the compounds required for life and sustenance. Our body can only thrive if we have the required nutrients to carry out our basic necessary biology. Simple logic suggests that a deficiency of essential raw nutrient materials precludes the ability of our body to make what it needs to undertake the required physiological processes of daily life—resulting in malfunction of the human machine and clinical sickness. There is an abundance of recent evidence in the scientific and laboratory literature expounding on the consequences of nutritional deficiency [36].

One could hardly imagine a student of architecture graduating from a reputable school without comprehensive knowledge of building materials, how such materials are used, how to detect problems, and how to correct irregularities. If detailed knowledge of nutritional biochemistry is so fundamental to the practice of health care, why has instruction on nutritional status assessment and nutritional remediation not been taught in most medical schools? [37]. Many in the health science community have assumed that nutritional compromise cannot be a common determinant of illness in the developed world because they believe that most people are “getting all they need in their diet.” Accordingly, it has not seemed prudent to waste valuable time teaching nutritional biochemistry and clinical nutrition if this is not a common cause of ill-health.

**Figure 5:**
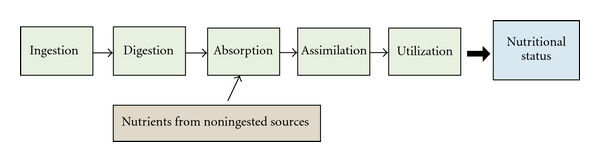
Determinants of Nutritional Status.

The fundamental flaw in this assumption is that nutritional status is not the same as food intake. Nutritional status commences with ingestion, but requires digestion, absorption, and assimilation—dysfunction occurring anywhere along the chain can result in metabolic compromise and disordered biology (Figure 5). Furthermore, some essential nutrients are primarily derived from noningested sources, such as vitamin D from sun exposure and vitamin K_2_ from enteric organisms. Emerging research confirms that significant nutritional deficiency of required materials is much more common than recognized and a ubiquitous cause of sickness [38]. Examples include vitamin D and some required lipids, recognized regulators of hundreds of genes, which have been found in several recent epidemiological studies to be deficient in many population groups [39, 40].

To demonstrate the clinical and public health significance of addressing deficiency states, it is illuminating to comparatively consider the projected benefit of diminished morbidity and mortality associated with widespread “national bowel cancer screening programs” versus maintenance of optimal nutrient status. Recent projections suggest that early detection methods and screening will reduce colorectal cancer mortality in those screened by 12–17% over the next 20 years [41]—a figure considered to be of notable significance when applied to large-scale populations. On the other hand, a large prospective study of colon cancer risk based on levels of 25(OH)D was published in *Lancet. *Assessing more than 25,000 participants, there was a 75–80% reduction in risk of ever developing colon cancer for those with higher levels of 25(OH)D compared to those with low levels [42]. Incorporation of a vitamin D strategy might yield favorable outcomes that far exceed any bowel cancer screening program; the profound health impact of remediating deficient biochemistry is evident.

### 4.5. Toxicity as a Cause of Illness

Since the Second World War, a chemical revolution has emerged in an effort to provide enhanced convenience, efficiency, beauty, comfort, and safety [43]. Affecting many aspects of our everyday lives, myriad synthetic chemicals are increasingly being found in our foods, our air, our water, and our bodies. Recent population studies by the Centers for Disease Control [44] as well as cord blood research undertaken by the American Red Cross [45] confirm widespread toxicant bioaccumulation in men, women, children, and the developing unborn. Research to understand and address the impact of these compounds on human health has confirmed that accrual of various toxic agents has become a widespread cause of disease [46]. At minute levels, toxicant compounds have potential to influence critical biological function in many ways such as by hormone disruption, immune dysregulation, cell damage, genetic influence, allergy induction, liver compromise, and cancer promotion [47]. Numerous afflictions, ranging from congenital malformations to cancer to hormonal irregularities, have recently been linked to adverse toxicant exposures [46]. 

Agents and forces that are toxic to the human body do not only include adverse chemical compounds but also encompass other determinants including biological agents [48, 49], physical toxicants [50], metabolic irritants [51], excessive psychological stress [52], and triggers for hypersensitivity or allergic reactions [53] (Table 1). As these different stressors can coexist, it is crucial to explore the total load or total body burden of adverse factors that may be causing illness. With history and emerging science confirming toxicants as a cause of sickness, why has this field been ignored for the most part in contemporary medical education?

As physicians are no longer bribed to poison business or political rivals as occurred in Hippocrates' day, it is assumed that patients are not regularly being exposed to significant levels of toxic agents. So why spend considerable time in medical school dealing with a nonexistent problem? This assumption is misguided, however, as evidenced by a plethora of recent medical literature expounding on exposure and bioaccumulation of toxic exposures as common etiological sources of illness. Many health bodies such as the World Health Organization have recently instituted programs to educate health practitioners about this growing concern [54, 55]. Emerging techniques and interventions to diagnose and eliminate accrued or persistent toxicants can have a profound impact on human health [56].

## 5. Quo Vadis: Science-Based Medicine

With the realization that irregularities in the modifiable environment of our bodies are the source cause of most chronic illness, the choice to change correctable factors can transform individual destiny. Health providers can facilitate health by uncovering factors responsible for disease and advising on a path to prevent illness and restore health [18]. Perhaps at some juncture in the future, technology will deliver humanity to a place where therapeutic epigenetic interventions will be used to suppress pathological genetic expression; at this point in history, however, addressing disease etiology still remains the best opportunity to prevent and overcome chronic affliction.

Is this alternative medicine? Hardly. It is scientific medicine based on perspicacious understanding of medical history, biochemistry, toxicology, infectious disease, immunology, and other mainstream scientific disciplines [18]. Finding out what is causing illness is fundamental to logical scientific medicine. One might expect a mechanic to find the cause of the knock in your engine; patients should expect at least as much from their doctors. Originating from the Latin verb “docere” meaning to teach, the term “doctor” might appropriately describe a trained scientist who educates patients on the cause of their illness and empowers them with instruction on solutions to prevent and overcome health afflictions.

Based on the tried-and-true model of clinical medicine—history, physical, laboratory investigations (including detailed nutritional status and toxicological assessment)—source causes of illness can be discovered and interventions to prevent and address molecular and biochemical irregularities can be implemented. Preventing birth defects by securing adequate folic acid [57], relieving post-partum depression by correcting fatty acid deficiency [40], restoring mental health by eliminating stockpiled toxicants [58–60], reversing some cases of autism by removing incitants and addressing nutritional deficiencies [22, 61], treating pediatric arthritis by managing food intolerances [62], overcoming impairment resulting from some chromosomal anomalies by remediating biochemistry [63], resolving inflammatory bowel disease with avoidance of sensitivities [53], relieving asthma and chronic fatigue by mold remediation [48], ending the tragedy of habitual abortion by addressing electromagnetic toxicity [50], and the author's experience of achieving remission from leukemia in a patient by eradicating retained aflatoxin are all examples of what can possibly be realized if underlying causes of sickness are explored, identified, and properly managed. 

There are some who feel that change in health care may soon happen. Von Eschenbach, a former Commissioner in the US Food and Drug Administration recently stated that “[the] transformation… is so profound and so radical that I call it a metamorphosis: a molecular metamorphosis in which the future of health and healthcare will be no more like the past than a butterfly is like a caterpillar… it will alter and change not just one thing; it will change everything” [64]. Some are not so optimistic. It is well recognized historically that progress along the path to change in health care proceeds lethargically and often occurs only after education and empowerment of subsequent generations [65–70]. Many researchers, clinicians, and health administrators of each epoch steadfastly refuse to consider iconoclastic evidence, no matter how compelling; some remain immune to the power of facts—no matter how true, no matter how precise. Indeed, when considering the actualities of evidence-based medicine, the stark reality of trying to persuade clinicians to open their minds to evidence contrary to entrenched beliefs and practices has been likened to the challenge of “teaching old dogs new tricks” [66], leaving some pioneers wondering whether medicine is more about ideology and religion than science [68]. As a result, knowledge translation remains notoriously slow which accounts for Nobel Prize winner Max Planck's sobering observation that science progresses funeral by funeral [71]. 

In a one-week course in medical school, however, it would be possible to convey the necessary information to competent medical trainees in order to establish the required foundations to investigate and manage patients presenting with chronic illness and to educate aspiring public health candidates to implement programs to prevent illness in population groups. In an era doused by the chemical revolution, medical students need to learn how to explore toxicant categories and to acquire clinical skills to investigate for and eliminate toxic factors. Instruction in nutritional and metabolic biochemistry with practical clinical applications is fundamental to competent medical practice. If a patient is depressed, is there a problem with her/his serotonin pathway such as tryptophan deficiency? Does she/he lack coenzymes or cofactors required to convert tryptophan to 5-HTP and then to serotonin? If so, why, and what can be done about it? If chronic metabolic dysfunction is clinically apparent, is there an acquired error of metabolism because of a toxicant induced enzyme malfunction? What do the laboratory results from the urinary organics testing demonstrate? This is science. The reflexive “have an ill, pop a pill” approach to chronic illness without investigating etiology is hardly consistent with perspicacious medical or clinical science.

## 6. Concluding Thoughts

Since the dawn of civilization, humankind has sought to explain the phenomenon of illness and affliction. Why is it that one person enjoys robust health, while another suffers? What is it that transforms a healthy individual from vigor and vitality to pain and chronic disability? Rather than the fatalistic outlook of genetic destiny, ongoing scientific evidence confirms that virtually all illness commences because of modifiable environmental causes, it persists because such environmental causes persist, and it can only abate when such causes are addressed. 

So, frankly, why do we get sick? The evidence shows that, although there are myriad ways in which people manifest sickness, there are only a limited number of ways in which people get sick. People get sick because of vulnerable genetics interacting with potentially modifiable factors in their environment. What are these changeable environmental determinants? The expanding body of scientific research in epigenetics, environmental health, and molecular medicine verifies what medical history has repeatedly and consistently confirmed—that deficiency and toxicity states cause disease. These two causative origins, however, remain blind spots in the contemporary approach to ill-health by much of the conventional medical community. Remediation of deficient biochemistry and elimination of toxicants has enormous potential to preclude chronic illness and restore health. Rather than fashionable wisdom that may be obsolete a year or a generation from now, these observations about disease origins and the associated clinical approach represent the accumulated wisdom of cutting-edge medical science through the ages.

## Figures and Tables

**Table 1:** Categories comprising the total body burden of potential toxicants.

(1) Chemical toxicants—for example, heavy metals, mycotoxins, and so forth	
(2) Biological toxicants—for example, viral agents, fungal exposures, and so forth	
(3) Physical toxicants—for example, radiation, trauma, and so forth	
(4) Metabolic toxicants—for example, hyperinsulinemia, elevated uric acid, and so forth	
(5) Psychological toxicants—for example, inordinate chronic stress, abuse, and so forth	
(6) Hypersensitivity toxicants—for example, intolerances such as peanut allergy, and so forth	
